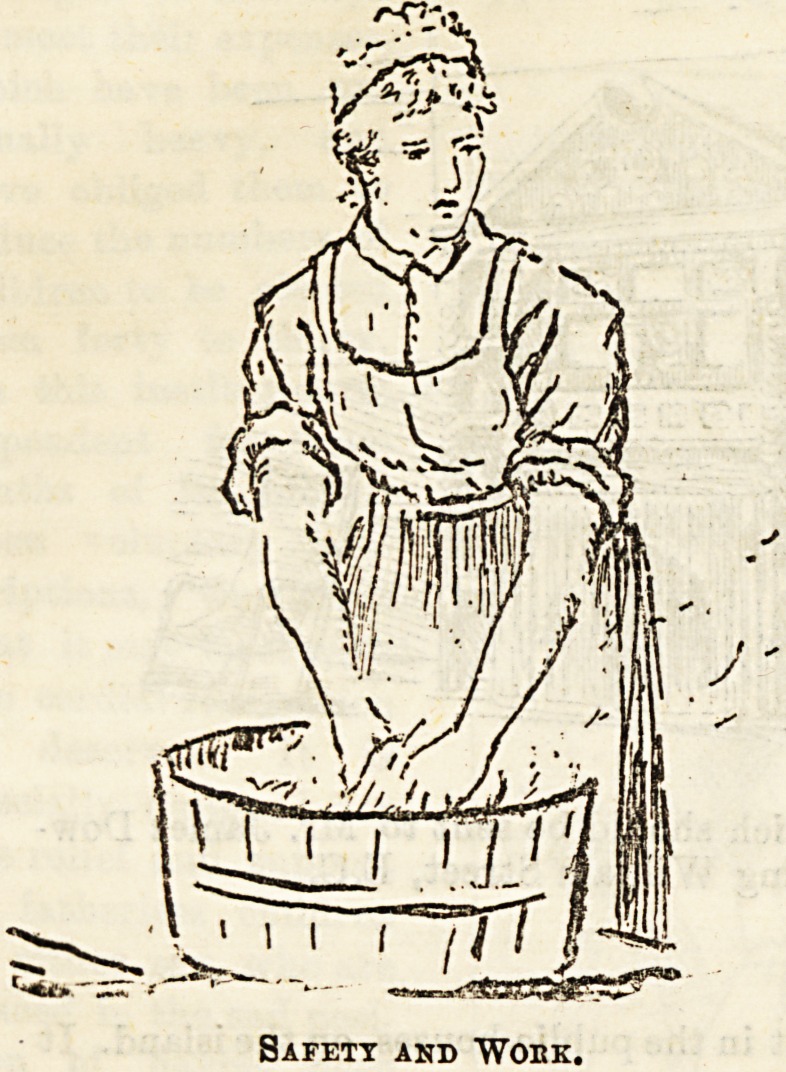# Miscellaneous Special Hospitals

**Published:** 1891-01-03

**Authors:** 


### MISCELLANEOUS SPECIAL HOSPITALS.

**Lock Hospital and Asylum,** Harrow Road, W.— If it were more generally known what a good work of rescue is being done by means of this hospital and asylum, we feel sure that it would meet with the support it deserves. The result, that considerably more than one-fourth of all those that pass through this hospital are reclaimed from their evil lives, points to a great work, which should be far more encouraged. It is not sufficiently realised by many what an important influence may be exerted over the mind of a patient during the period of bodily suffering. It is sought to exercise an influence for good from the first moment a patient enters the hospital, and so to lead to better aims to rescue the sufferer from a life of misery. As soon as a patient makes up her mind to go into the Lock Asylum, she is removed altogether from the presence of former companions, is placed in a different part of the building, where she may have a chance of setting herself entirely free from her former life, and where useful occupations are given her (amongst others, excellently-managed laundry work), so that after a time, spent under the careful training of the matron, she enters a new life of useful work. Secretary, Mr. A. P. Coote, M.A.; Matron, Miss Kydd.

SAFETY AND WORK.

**London Skin Hospital,** 47, Cranbourn Street, Leicester Square, W.C.—Only out-patients are seen to at this hospital, owing to the funds at the disposal of the Managers not allowing them to treat in-patients. Secretary, Mr. H. A. Gifford. Help is much needed.

**National Hospital for Diseases of the Heart,** 32, Soho Square.—In-patients in this hospital are admitted on the recommendation of a life-governor, but a limited number of deserving cases are admitted on payment of 10s. a week, and expenses. In order to keep a larger number of beds available for this latter class of patients, funds are earnestly appealed for. Matron, Mrs. Roberts; Secretary, Capt. F. Handley.

**Royal London Ophthalmic Hospital,** Blomfield Street, E.C.—Contributions are much needed in support of this, the oldest ophthalmic hospital in Great Britain. Some 27,000 out-patients attend annually, and the one hundred beds were occupied by 2,337 patients last year. Secretary, Mr. A. J. Newstead; Matron, Miss Nichol.

## Figures and Tables

**Figure f1:**